# A structural group-connectome in standard stereotactic (MNI) space

**DOI:** 10.1016/j.dib.2015.08.035

**Published:** 2015-09-07

**Authors:** Andreas Horn

**Affiliations:** Charité – University Medicine, Dpt. for Neurology, Clinical Research Group 247, Movement Disorders Section, Department of Neurology, Charité – University Medicine (CVK), Berlin, Germany

## Abstract

A group connectome of 20 subjects has been normalized into standard stereotactic (MNI) space. Data has been processed using the Gibbs' Tracking approach (Reisert et al., 2011) [11] and normalized into standard space using DARTEL (Ashburner, 2007) [1]. All data has been acquired within the scope of the study A. Horn, D. Ostwald, M. Reisert, F. Blankenburg, The structural–functional connectome and the default mode network of the human brain, NeuroImage 102 (2013) 142–151. http://doi.org/10.1016/j.neuroimage.2013.09.069. The utility of this dataset can be described by the following points: In medical studies in which subject-specific dMRI is not available, a standardized connectome may help to gain some canonical insight into white-matter connectivity. The dataset enables scientists who use different modalities (like EEG, MEG etc.) without access to MRI, to combine studies obtained using other methodology with insights from the brain's inner structural formation. The dataset could also extend possible claims made by meta-analyzes/literature-based studies.

Specifications Table

**Table:** 

Subject area	Neuroscience
More specific subject area	Connectomics
Type of data	Fiberset of structural pathways of the human brain
How data was acquired	Diffusion-weighted MRI
Data format	MATLAB/Trackvis
Experimental factors	Gibbs' fiber-tracking, DARTEL normalization
Experimental features	The dataset can be used to perform fiber-tracking in terms of a fiber-selection within standard stereotactic MNI space.
Data source location	Berlin, Charité – University Medicine, Berlin Center for Advanced Neuroimaging (BCAN)
Data accessibility	Data is downloadable without restriction using the following URL:www.lead-dbs.org/?page_id=23

**Value of the data**

• Canonical template of the white-matter architecture of the human brain.
• Perform fiber tracking within standardized MNI space.
• Study structural connectivity of normal brain anatomy.

## 1 Data, experimental design, materials and methods

**Fig. 1:**
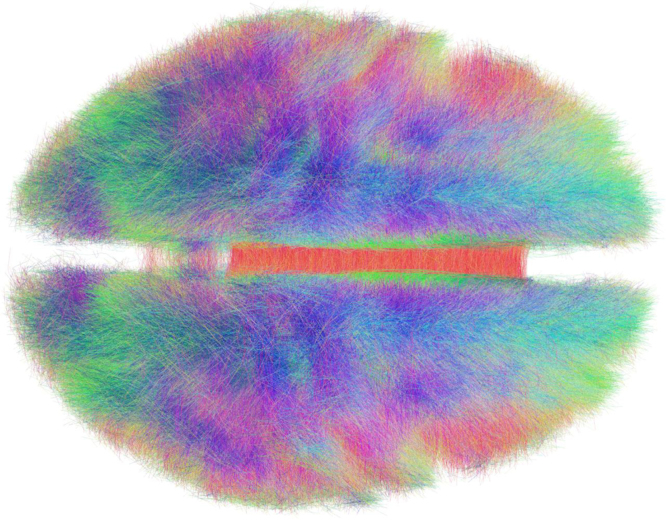
Rendering of the group connectome based on 20 subjects. 3% of all estimated fibers are shown, color-coded by traversing direction (*xyz*-directions mapping to rgb colors respectively). Visualization of fibers was done using TrackVis software (www.trackvis.org).

A previously published tractography dataset from 20 normal subjects was used to establish the group-connectome in standard stereotactic (MNI) space [9]. For details of this dataset and tractography (pre-)processing, please refer to the primary publication [9]. Briefly, MRI data including diffusion-weighted images were collected via a single-shot spin-echo planar imaging sequence (TR=10,000 ms, TE=94 ms, 2×2×2 mm^3^, 69 slices) from 20 healthy subjects. An effective *b*-value of 1000 s/mm^2^ was used for each of 61 diffusion-encoding directions. The current dataset includes data from one additional subject, a 27-y-old male that was not included in the original publication because of artifacts in fMRI sequences (diffusion data were artifact free). Diffusion data from each subject were processed using the global Gibbs' tracking approach [11] using standard parameters. This approach models data based on properties of a whole-brain fiber set that is subsequently compared and optimized to the whole-brain diffusion MRI data in an iterative optimization routine. The approach was top performer in a blinded comparison of different tractography processing algorithms [4]. Connectivity data from each subject then were normalized to MNI space using a diffeomorphic image registration algorithm as implemented in SPM8 ([1]; www.fil.ion.ucl.ac.uk/spm/) to create a single group-level connectivity dataset. The resulting dataset is available in Matlab (http://www.mathworks.com/) and TrackVis (http://trackvis.org) formats and can be used to perform ‘fiber-tracking’ in form of a post-hoc fiber-selection of fibers from the whole-brain dataset. It can be directly used to perform analyzes in LEAD DBS (www.lead-dbs.org; Matlab; [13]) and TrackVis software. Fig. 1 shows 3% of fibers stored within the dataset.

**Fig. 2:**
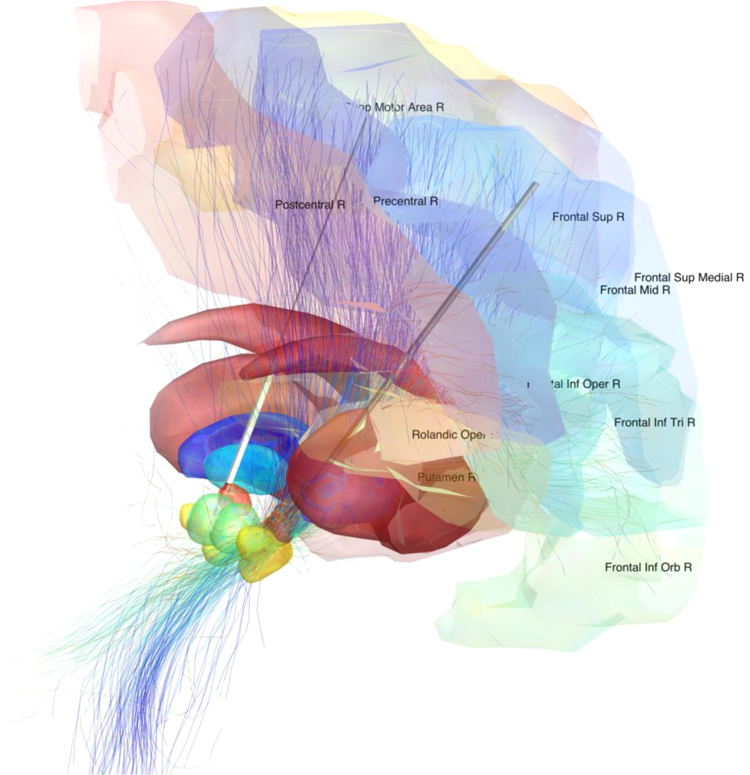
Example application of the group connectome. Deep brain stimulation electrodes of a patient suffering from Parkinson's Disease were reconstructed based on post-operative structural MR imaging. The volume of tissue that was stimulated (VAT) was estimated based on a model by Mädler and Coenen [6] and the actual stimulation settings of the patient's implanted pulse generator. Fiber tracts that traversed through the VAT were selected from the group connectome. Areas that were connected to the VAT by selected fiber tracts were selected from the automated anatomic labeling (AAL) atlas [12] and visualized. In addition to AAL regions, subcortical nuclei from the ATAG-atlas [10] are visualized: striatum (red), external part of the pallidum (blue), internal part (cyan), subthalamic nucleus (orange), red nucleus (green), substantia nigra (yellow). Analyzes and visualization were performed directly within MNI space using LEAD-DBS software (Horn, 2015; www.lead-dbs.org).

The following non-exhaustive list of potential use-cases may illustrate the utility of the data set:

1. In medical studies where subject-specific dMRI is not available and cannot be acquired, a standardized group-connectome might help to gain at least some canonical insight into white-matter connectivity if the interpretation of results is done carefully. A direct example is the field of deep brain stimulation (DBS), where patients are subject to a very limited signal absorption rate (SAR) and thus dMRI can only be acquired with many limitations postoperatively. However, in DBS, electrode locations can be precisely modeled (Horn, 2015), and their spatial relationship to fiber-tracts can lead to profound insight into the mechanism of action of DBS [2,3]. An example use case is illustrated in Fig. 2.
2. The dataset may enable scientists working in different fields of research (e.g. in machine-learning) and use different methodologies (e.g. EEG, MEG), to enhance their studies with insights from the brain's inner structural architecture.
  The dataset could also extend possible claims made by meta-analyzes/literature-based studies (e.g. see Fox et al. (2014) [5], where this connectome has already been applied).
3. Last, in fMRI imaging, it is common to use extensive spatial smoothing to account for inter-subject anatomical variation when pooling over subjects [8]. This group-connectome may yield potential to do so more elaborately, i.e. based on structural connectivity – as has been done based on functional imaging [7].

Taken together, a whole-brain structural group connectome based on 20 healthy subjects that can be used to perform ‘fiber tracking’ in terms of a post-hoc fiber selection directly within MNI-space is released alongside this publication. The dataset can be used to study the white matter architecture of the human brain, as a ‘reference connectome’ or to study particular white matter tracts within a well-characterized and widely applied standardized stereotactic space.
